# The M-band: The underestimated part of the sarcomere^☆^

**DOI:** 10.1016/j.bbamcr.2019.02.003

**Published:** 2020-03

**Authors:** Stephan Lange, Nikos Pinotsis, Irina Agarkova, Elisabeth Ehler

**Affiliations:** a Biomedical Research Facility 2, School of Medicine, University of California, San Diego, Medical Sciences Research Bldg, 9500 Gilman Drive, La Jolla, CA 92093-0613C, USA; b University of Gothenburg, Wallenberg Laboratory, Department of Molecular and Clinical Medicine, Institute of Medicine, Gothenburg, Sweden; c Institute of Structural and Molecular Biology, Department of Biological Sciences, Birkbeck College, Malet Street, London WC1E 7HX, UK; d InSphero, Wagistrasse 27, CH-8952 Schlieren, Switzerland; e Randall Centre for Cell and Molecular Biophysics, School of Basic and Medical Biosciences, King's College London, New Hunt's House, Guy's Campus, London SE1 1UL, UK; f School of Cardiovascular Medicine and Sciences, British Heart Foundation Research Excellence Centre, King's College London, New Hunt's House, Guy's Campus, London SE1 1UL, UK

**Keywords:** Cytoskeleton, Sarcomere, Cardiomyopathy, Myomesin, Obscurin

## Abstract

The sarcomere is the basic unit of the myofibrils, which mediate skeletal and cardiac Muscle contraction. Two transverse structures, the Z-disc and the M-band, anchor the thin (actin and associated proteins) and thick (myosin and associated proteins) filaments to the elastic filament system composed of titin. A plethora of proteins are known to be integral or associated proteins of the Z-disc and its structural and signalling role in muscle is better understood, while the molecular constituents of the M-band and its function are less well defined. Evidence discussed here suggests that the M-band is important for managing force imbalances during active muscle contraction. Its molecular composition is fine-tuned, especially as far as the structural linkers encoded by members of the myomesin family are concerned and depends on the specific mechanical characteristics of each particular muscle fibre type. Muscle activity signals from the M-band to the nucleus and affects transcription of sarcomeric genes, especially via serum response factor (SRF). Due to its important role as shock absorber in contracting muscle, the M-band is also more and more recognised as a contributor to muscle disease.

## Highlights

• This article reviews the structure, composition and function of the M-band, the central structure of the sarcomere, the basic unit of a myofibril.
• In addition, signalling pathways emanating from the M-band, which may affect protein turnover from triggering transcription (via SRF) to promoting degradation are discussed.
• A particular strength of the review is the links that it provides throughout the text to disease.

**Fig. 1:**
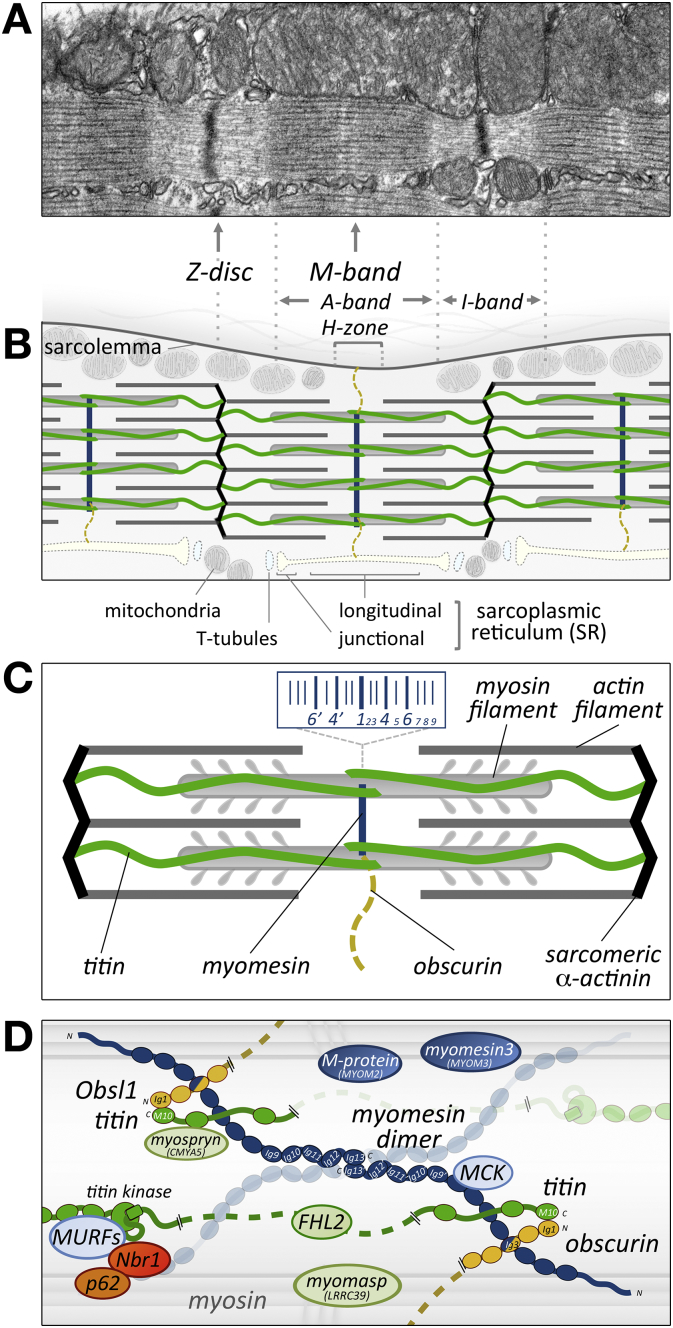
The M-band in the sarcomere. A) Electron micrograph of longitudinally cut cross-striated muscle showing myofibrils (bottom half) and mitochondria (top half). Sarcoplasmic reticulum (SR) is located alongside the myofibrils. B) Schematic representation of A. C) Schematic representation of a sarcomere, the basic unit of a myofibril. Thin (actin and associated proteins) and thick (myosin and associated proteins) filaments are depicted in grey; the Z-discs are shown in black, titin filaments are green, the M-band is shown in blue and obscurin is shown in yellow. The inset indicates the arrangement of the M-lines, which are substructures of the M-band. D) Model of the molecular arrangement of major M-band components. Members of the myomesin family are shown in blue, but only myomesin is depicted as a molecule, forming dimers that link neighbouring myosin filaments (grey). Titin is shown in green and obscurin in yellow. Associated proteins such as M-CK, FHL2, Nbr1, p62, myospryn and myomasp are only indicated as ellipses, not as molecules.

The paracrystalline arrangement of the contractile proteins actin and myosin in cross-striated muscle is due to two transverse structures in the sarcomere, the basic unit of the myofibrils, which are the Z-disc and the M-band (Fig. 1). The structural and signalling role of the Z-disc is studied widely and its components and role are reasonably well understood and frequently reviewed (e.g. [[1], [2], [3]]). The M-band has received much less attention and the most recent reviews solely dedicated to this structure were published somewhat out of the limelight [4,5]. Aim of this article is to put this crucial sarcomeric element into the spot light, focusing on its molecular composition, ultrastructure and response to mechanical challenges as well as to speculate on its function in muscle.

The M-band is located in the middle of the sarcomere and serves to arrange the thick filaments into the A-bands (Fig. 1A, B). The members of the myomesin gene family (myomesin [6] encoded by MYOM1 in humans, M-protein/myomesin-2 [7] encoded by MYOM2 and myomesin-3 [8] encoded by MYOM3) are believed to act as structural linkers between the thick filaments. They share a common domain arrangement with an intrinsically unstructured head domain, followed by twelve immunoglobulin (Ig; belonging to the I-set [9,10]) and fibronectin type III (Fn) domains [8,11,12]. Myomesin itself is constitutively present in the M-bands of all striated muscles in a nearly stoichiometrical ratio to sarcomeric myosin [13], while the two other proteins are differentially expressed in muscle types. Textbook images tend to show electron micrographs of longitudinally sectioned fast twitch fibres of skeletal muscle (e.g. human tibialis anterior), because they show the most ordered version of this structure. In these images it is possible to discern the substructures of the M-band, called the M-lines, which are arranged symmetrically across the very centre of the sarcomere ([14]; inset in Fig. 1C). Depending on muscle type (fast twitch, slow twitch, cardiac), developmental stage and species, the numbers of M-lines will vary, with only M4 (and M4′) being consistently prominent [15]. It is thought that the consistent visibility of M4 is due to M-CK (muscle isoform of creatine kinase) decoration of the M-lines [16]. Contrary to erroneous beliefs still purported by some that the absence of an electron dense structure in electron micrographs might mean the lack of an M-band [17], if assessed from a molecular point of view, M-band proteins such as myomesin can be detected as soon as the first A-bands can be distinguished during myofibrillogenesis [18].

## 1 What is the role of the M-band?

**Fig. 2:**
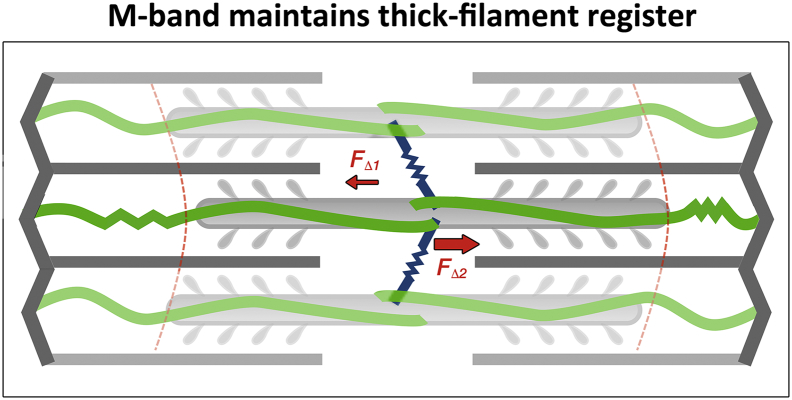
M-band maintains thick filament register. A scheme showing how force imbalances are suggested to lead to a misalignment of thick filaments, which then get realigned by contributions from titin and from structural components of the M-band such as myomesin.

Classical investigations on the sarcomere by Squire and colleagues in the last decades of the 20th century, established the M-band as the structure that cross-links the myosin filaments into a hexagonal lattice and defines their relative rotations around their long axes [15,19,20]. The mechanical stability of the M-band network is achieved through specific architectural features that are unique to this compartment. Higher resolution electron micrographs of the sarcomere display a bare zone around the M-band, which is due to the lack of myosin heads. As the myosin filaments overlap towards the centre of the sarcomere, five major symmetrical placed non-myosin densities are clearly observed in fast skeletal muscle. These are mostly referred to as M-lines or M-bridges, namely M6′, M4′, M1, M4 and M6 and they follow a trigonal symmetry. Additional peaks have been also been observed (e.g. M3-M3′, M8-M8′ and M9-M9′) followed by three myosin crown levels at the M-band periphery [21]. Based on these structural observations, Agarkova and Perriard proposed the crucial role of the M-band for the stability of the activated sarcomere at the beginning of this century [22]. A system constructed of only actin and myosin filaments is intrinsically unstable in the longitudinal direction, as the forces that are produced by activated myosin heads are not exactly the same on the left and right halves of the bipolar myosin filament, and any deviation from the central position will increase the imbalance (Fig. 2). The titin filament is a rather weak spring, which is not able to counteract the forces generated even by few myosin heads. However, a system that contains a higher number of myosin filaments connected in the middle with short linkers will be more stable, as stochastic differences between adjacent filaments can be averaged by the elastic web of the M-band filaments [22]. As the vertebrate sarcomere contains about 1000 myosin filaments, the M-band reduces the longitudinal instability by about 30 times (square root of 1000). Thus, the M-band absorbs the misbalances of active forces through the myosin filament lattice and aids titin in keeping the central position of the A-band in the sarcomere. Playing the role of a shock absorber, the M-band filaments can be subjected to strong mechanical forces during sarcomere contraction, and may even be ruptured in extreme cases, as detected in electron micrographs [23].

## 2 M-band composition is fine-tuned depending on developmental stage, fibre type and species

**Fig. 3:**
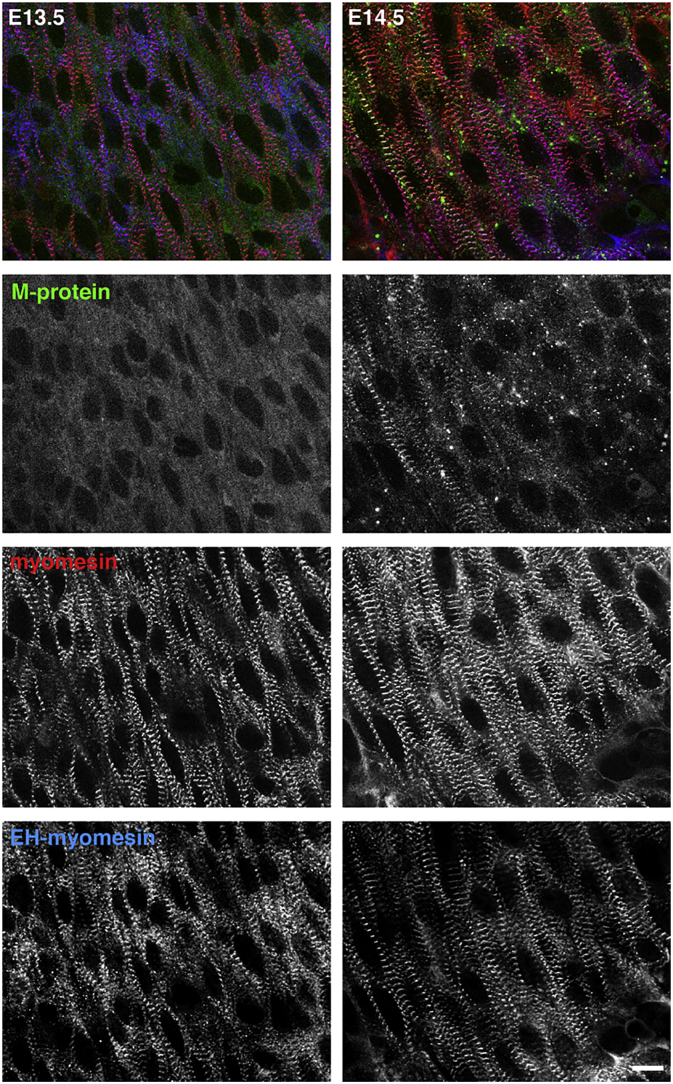
M-protein expression is only upregulated later during heart development. Confocal micrographs of mouse embryonic heart whole mount preparations from embryonic day 13.5 (left column) and embryonic day 14.5 (right column) immunostained with antibodies against M-protein (second row; green in overlay), myomesin (third row; red in overlay) and EH-myomesin (blue in overlay). Striations for M-protein only start to appear in some of the myofibrils at E14.5. Scale bar = 10 μm.

Isolated fragments of myosin tails can assemble to helical filaments, with the assembly mode being determined by 195 residue segments along the tail [24]. For their integration to the bipolar filaments that constitute the A-band it is assumed that also myomesin and the C-termini of titin are required [18]. Based on antibody epitope localisation of major M-band proteins in electron micrographs, more than twenty years ago a model was proposed that shows the C-terminal ends of titin to overlap in an antiparallel fashion and it was suggested that myomesin provides a link between myosin and titin [25], also based on its ability to bind both in biochemical assays [26]. This model was refined ten years later, when it was discovered that myomesin in the M-band is actually an antiparallel dimer, with its C-termini forming a tight interaction interface and its N-termini anchoring it to myosin ([27,28]; see Fig. 1D). The three molecules myosin, titin and myomesin are still thought to be the minimal requirement for the assembly of an A-band during myofibrillogenesis [18]. Depending on muscle fibre type, this minimal M-band structure gets reinforced by the incorporation of the two other myomesin family members. Sarcomeres in muscle types that are exposed to higher forces (e.g. fast twitch, cardiac in mammals) tend to express M-protein, which also binds to myosin and titin in biochemical assays [29,30]. Muscles that express M-protein have a well-defined ultrastructure in electron micrographs with prominent M1 lines, which gives rise to a model that M-protein makes up a perpendicular connection between adjacent myosin filaments in the very middle of the sarcomere [25]. Sarcomeres in slow fibres and in embryonic heart on the other hand often lack clearly discernible M-bands in electron micrographs, which is most likely due to the presence of a splice variant of myomesin, EH-myomesin (embryonic heart), which bears an elastic EH-domain in the middle of the molecule [31]. The elasticity that the EH-domain confers to myomesin [32] may be required in muscle types that undergo eccentric contractions such as slow twitch, extraocular and embryonic heart muscle [13,31,33]. The first M-bands in the embryonic heart lack M-protein and its expression is only switched on at later fetal stages (e.g. embryonic day 14.5 in the mouse; Fig. 3). Around birth the expression of EH-myomesin gets downregulated in heart and M-protein gets upregulated [4,31]. The third member of the myomesin family, myomesin-3, is expressed mainly in intermediate speed fibres of skeletal muscle (type IIA), but its expression can also be detected in human heart [8,34].

The origin of myomesin is linked to the evolution of striated muscle and the establishment of different muscle types. The genome of the lancelet, the modern representatives of the ancient chordate lineage, contains only one myomesin gene, and consequently, the sarcomeres demonstrate only M4 and M4′ lines on electron micrographs [35]. A whole-genome comparison of the three chordate groups (tunicates, lancelets and vertebrates) has indicated that the vertebrate lineage arose as a result of two genome-wide duplications and subsequent reorganisations. Therefore, the myomesin protein family appeared probably as a result of two subsequent duplications of the original common myomesin ancestor gene and the following diversification into the different muscle fibre types.

The specific expression pattern of different myomesin family members depending on muscle type and developmental stage (for a scheme in mouse see [4]) is a useful tool for assessing the maturity for cardiomyocytes derived from human embryonic stem cells or induced pluripotent stem cells (iPSC). In electron micrographs of differentiated human iPSC-cardiomyocytes clear M-band structures could only be distinguished after 360 days in culture, which correlated well with the increased expression of M-protein as detected by qPCR [36]. Immunofluorescence analysis of differentiated human iPSC-cardiomyocytes also suggested a rather immature M-band status with high levels of expression of EH-myomesin but little or no expression of M-protein [37]. In our hands, M-protein positive M-bands can be detected after long-term culture in 2D (>60 days) or when the cells are cultured in 3D, which tends to promote their maturation (Ormrod and Ehler, unpublished). In conclusion, the expression patterns of different members of the myomesin family correlate with the appearance of the M-band in electron micrographs and with distinct functional properties of muscle types.

## 3 Alterations in M-band composition in disease

If the expression of different myomesin family members is so specific for a muscle type, it should also be responsive to changes in demand, for example in disease. Indeed, several transcriptomics and proteomics studies showed that up- or downregulation of especially the expression of myomesin (MYOM1) and its isoforms accompanies the response of a muscle to varying kinds of stress in myopathy [[38], [39], [40]]. In addition, re-expression of EH-myomesin was reported to be a hallmark of dilated cardiomyopathy (DCM) in mouse models for the disease as well as in human patients [34]. At the moment it is unclear, whether this upregulation of EH-myomesin is an adaptive response to improve the stability of sarcomeric structure in conditions of eccentric contractions or whether it is maladaptive due to reduced contractile force produced by less aligned contractile filaments. While in some patient populations a direct correlation of EH-myomesin expression levels and ventricular dilation was found, with a potentially inverse correlation of EH-myomesin expression levels and ejection fraction [34], in other patient populations a positive correlation between left ventricular ejection fraction and EH-myomesin levels was detected (Pluess and Ehler, unpublished). The situation gets even more complex when different subtypes of DCM are analysed, since EH-myomesin expression is upregulated in almost all subtypes but not in patients with peripartum DCM [41], which often have an even worse prognosis. In one mouse model for DCM, the MLP knockout mouse [42], almost all the cardiomyocytes in the heart are positive for EH-myomesin and also the expression of M-protein is maintained completely [34]. The MLP knockout mice have a normal life span [43], while another mouse model for DCM, that is caused by overexpression of beta-catenin (delta exon 3 mice; [44]) does not survive beyond five months. These mice only partially upregulate EH-myomesin expression and also show a loss of M-protein expression in a subset of cardiomyocytes [34]. The situation is further complicated by the observation that hearts from MLP-knockout mice start to express myomesin-3, while hearts from delta exon 3 mice fail to upregulate its expression [34]. This suggests that whether a genetically modified mouse model can tolerate a DCM-causing insult or not may depend on how many myomesin family members there are in an M-band. In human DCM, M-protein and myomesin-3 expression levels appear to be constant and it is only EH-myomesin that gets upregulated [34]. Ultimately it may need a mouse model that consistently expresses EH-myomesin in the adult and a detailed analysis of its M-band composition and physiology to answer this question.

The re-expression of EH-myomesin in DCM is a consequence of altered alternative splicing, which is a common response in cardiomyopathy (reviewed in [45]). Especially the RNA binding motif proteins 20 and 24 (Rbm20, Rbm24) appear to be major players in alternative splicing in striated muscle cells. For example, it was shown that a loss of function in Rbm20 in rats leads to persistently longer titin accompanied by chamber dilation and increased frequency of sudden cardiac death [46]. Rbm20 affects not only the splicing of titin but also of myomesin [47] and is among the most frequently mutated genes in DCM [48]. This differential splicing of titin and myomesin is also seen in iPSC-derived cardiomyocytes from a patient with the S635A mutation in the Rbm20 gene [49]. Knockout mice for Rbm24 display less distinct M-bands in electron micrographs from skeletal muscle, which could be rescued by the expression of GFP-tagged Rbm24, suggesting a direct effect [17]. The ultrastructure of the Rbm24 knockout skeletal muscle could be explained by a switch from M-protein to EH-myomesin expression, resulting in M-bands similar to slow twitch fibres, but unfortunately the molecular composition of the M-band, especially as far as myomesin family members is concerned, was not analysed in these mice. Conditional knockout of Rbm24 postnatally also leads to dilated cardiomyopathy in mice [50]. Interestingly the expression levels of Rbm20 and Rbm24 are repressed by hypertrophic stimuli and there is evidence for their cooperation [51]. This suggests that changes in alternative splicing of elastic proteins such as myomesin and titin contribute to a cardiomyopathy phenotype, especially in the case of dilated cardiomyopathy [34,52].

## 4 Other M-band associated proteins

Apart from the myomesin family members, which appear to be the major structural linkers in the M-band, several other proteins were found in this region of the sarcomere, most of them only in this century. M-CK, the muscle isoform of creatine kinase was shown to bind to central domains of both myomesin and M-protein [53] and is probably the cause for the electron dense signal that is picked up in electron micrographs as M-lines [16]. Mice that lack M-CK are viable but show impaired muscle function upon challenge, suggesting that M-CK at the M-band is required for optimal contractile response and physiological performance [54]. The C-terminus of titin interacts with obscurin and obscurin-like1 ([55], discussed below) as well as with myospryn [56], which is a large tripartite motif (TRIM) protein encoded by a gene associated with cardiomyopathy (CMYA5). The regulation of these interactions and whether they are competitive is not very well understood at present. Another protein that binds to an unstructured C-terminal region of titin known as the is2 region, is the Four and a Half LIM domain protein 2 (FHL2 or DRAL), which mediates the subcellular targeting of metabolic enzymes such as creatine kinase, adenylate kinase and phosphofructokinase [57]. The C-terminus of myosin binds Myomasp, which is a leucine-rich protein that may be involved in stretch sensing and appears to affect serum response factor (SRF)-dependent gene expression [58].

## 5 No longer so obscure M-band and membrane links

First images of links between the M-band and membranous structures in the cell were provided in electron micrographs [59]. Since then the molecular composition of these links has been elucidated in greater detail. A first indication of the molecular nature was provided with the discovery of obscurin, a giant protein with a similar architecture and domain layout to titin [60,61]. The gene for obscurin gives rise to at least three splice products: the giant obscurin-A and obscurin-B isoforms, and the kinase-only protein (KIAA 1639, sometimes also referred to as obscurin MLCK), which originates from a separate promoter [62,63]. Recent reports indicate the presence of additional splice isoforms, which are significantly smaller than the giant obscurin variants [64]. Obscurin itself consists mainly of serially linked Ig domains, which are interspersed with unstructured linkers and Fn domains. Similar to titin, obscurin harbours in its C-terminus signalling properties. However, while titin contains only one kinase domain, obscurin encodes two kinase domains in addition to a SH3-DH-PH domain triplet (acting as a RhoGEF) that associate obscurin with phospholipids in the membrane [64] and link it to Rho-dependent signalling [65], as well as an IQ-motif that was shown to bind to calmodulin [61]. The obscurin protein family contains two other members: obscurin-like 1 (Obsl1), which shows high similarity to the obscurin N-terminus, and the striated muscle preferentially expressed gene (SPEG), which exhibits homology to the obscurin C-terminus, including its kinase domains. All proteins in the obscurin family are thought to have originated from one ancestral gene [66], a hypothesis that is supported by the existence of only one obscurin orthologue in invertebrates, called UNC-89 [67]. Obscurin, Obsl1 and SPEG display wide-ranging tissue-specific expression patterns and subcellular localisations. While obscurin is primarily expressed in striated muscle tissues [68], recent studies indicate its expression in non-muscle tissues and a role for tumorigenicity and metastasis [69,70]. Similarly to obscurin, SPEG was found to exhibit tissue-specific expression patterns, with high expression in skeletal muscle, heart, aorta and brain [71]. Hence, depending on the splice-isoform and tissue, SPEG is also called aortic preferentially expressed gene (APEG-1) or brain preferentially expressed gene (BPEG). In contrast, to obscurin and Obsl1, SPEG localises to the junctional sarcoplasmic reticulum (SR) in muscle tissue, displaying a doublet that encloses the Z-disc in immunofluorescence images [72,73].

Obsl1 is expressed in a wide range of tissues and displays a complex splice-isoform pattern [66]. Accordingly, mutations in the more ubiquitously expressed Obsl1 gene were linked to the development of the 3M-growth disorder [74,75], while mutations in SPEG/APEG and obscurin were associated with the development of cardiac and skeletal myopathies [[76], [77], [78], [79], [80], [81], [82]].

Specific roles of the obscurin protein family in the M-band are largely restricted to obscurin itself and its close homologue, Obsl1. Both proteins are localised to the M-band of mature muscles by interactions with myomesin and the C-terminal Ig-domain M10 of titin [55,61]. Other subcellular localisations for obscurin and Obsl1 were reported [64], depending on the antibodies used and the maturity state of the muscle [61]. While not much is known about functions for Obsl1 in muscle, specific roles for obscurin in cardiac and skeletal muscle were investigated better. In addition to myomesin and titin, obscurin was shown to interact with slow muscle myosin binding protein-C (sMyBPC) as well as another region in titin, the Ig-domains Z9/Z10 located at the sarcomeric Z-disc [61,83]. Apart from its association with sMyBPC, obscurin may also harbour an as-of-yet unidentified binding site for myosin, as the invertebrate homologue UNC-89 via its SH3 domain was shown to bind to paramyosin [84]. This invertebrate-specific ‘headless myosin’ exhibits 36–38% sequence homology to the myosin rod-domain and is found in the tubular core of thick filaments. However, while *C. elegans* UNC-89 mutants display profound disruption of their myosin filament system [84], obscurin knockout mice show no overt disorganisation of their thick filaments in skeletal or cardiac muscles ([68] & unpublished observation). Another possibility is that the SH3 domain of obscurin may interact with another as yet unidentified protein containing an SH3 binding motif.

**Fig. 4:**
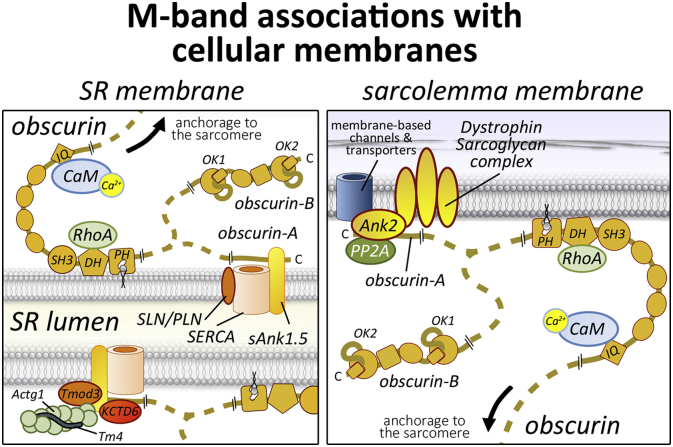
M-band associations with cellular membranes. Obscurin (depicted in yellow) mediates links between the M-band and the sarcoplasmic reticulum (left hand panel) as well as the plasma membrane (right hand panel).

Besides its interactions with sarcomeric proteins, the best characterised function for obscurin lies in the organisation of the sarcolemmal and sarcoplasmic reticulum (SR) membrane-architecture (Fig. 4). This function is achieved by binding of the unstructured obscurin-A C-terminus to various members of the muscle ankyrin protein family. The first of these interactions was reported for muscle specific small ankyrin-1 isoforms sAnk1.5 and sAnk1.9 [[85], [86], [87]], which lack all structural domains of classical erythrocyte-type giant ankyrin-1 isoforms (e.g. the tandem ankyrin-repeats). Instead, these small ankyrin proteins are largely unstructured, but contain a single transmembrane spanning region that is embedded in the SR-membrane of skeletal and cardiac muscle. Molecular studies on small ankyrin isoforms indicate their role for localising tropomodulin-3 (Tmod3) and gamma-actin (Actg1) to the SR [88], and in regulating the activity of the sarco(endo)plasmic reticulum Ca^2+^-ATPase (SERCA) calcium pump [89,90]. Experiments on cells or mice where obscurin was removed either by siRNA or gene-knockout validated that this interaction is required for the proper localisation and stability of sAnk1.5 at the sarcomeric M-band [68,87,91]. Loss of obscurin also resulted in dramatic architectural changes to the SR [68]. Knockout muscles displayed severely decreased amounts of longitudinal SR, which runs along the span of the sarcomeric A-band (Fig. 1B). Hence, the structural functions of obscurin/sAnk1.5 may be compared to a central ‘bridge pillar’, supporting the SR (‘bridge’) and connecting it with the underlying sarcomere (‘ground’) to provide stability to this membrane compartment. Loss of obscurin/sAnk1.5 leads to a collapse of the ‘bridge’ with resulting detrimental alterations to the SR architecture. Moreover, the role of obscurin as a linker between M-band and SR seems to be conserved in *C. elegans*: loss of function of UNC-89 results in disorganisation of the SR proteins, the calcium release channel (UNC-68) and SERCA [92].

Association of obscurin to ankyrin proteins is not restricted to small ankyrin-1 isoforms. Conserved obscurin binding domains were also found in B-type ankyrin (Ank2), and direct their localisation to M-bands in cardiomyocytes [93]. Giant ankyrin-B isoforms are membrane-associated proteins that require ankyrin repeats and spectrin binding for their membrane localisation and interaction with ion channels, transporters and cell-adhesion complexes, such as the dystrophin-sarcoglycan (DSG) complex. Due to their scaffolding functionality, these ankyrin isoforms coordinate the sarcolemmal-bound cytoskeleton in cardiac and skeletal muscles. Indeed, modulation of obscurin binding to ankyrin-B in cells did not only alter the M-band association of ankyrin-B itself, but also that of the closely bound protein phosphatase 2A (PP2A) [93]. These results indicate that obscurin may also play a role in the regulation and integrity of sarcolemmal membrane architecture (Fig. 4), a hypothesis that was confirmed when investigations in obscurin knockouts revealed disruptions to the localisation of dystrophin and the sub-sarcolemmal microtubule cytoskeleton [73,94]. In conclusion, obscurin and its relative UNC-89 seem to be important for providing a link between myofibrils and membraneous compartments of the cell in mammals as well as in *C. elegans*.

## 6 Signalling from and to the M-band

Little is known about muscle specific signalling around the M-band, despite the localisation of several protein kinases (kinase domains of titin and obscurin), phosphatases (e.g. PP2A), proteins involved in Rho-signalling (RhoA, obscurin) and in protein turnover (proteins of the muscle ring finger (MURF) family), as well as signalling adaptors (FHL2, myospryn) to this region of the sarcomere. Currently the best-characterised signalling cascade in the M-band region is the one that emanates from titin's kinase domain at the edge of the M-band [95]. After a conformational change that can be induced by mechanical stretch [96], titin kinase domain can bind to nbr1, a protein involved in autophagy in muscle cells [97]. Nbr1 in turn binds to p62, another scaffolding protein involved in autophagy, which then interacts with MURF2, a muscle specific ubiquitin E3 ligase. MURF2 binds to SRF, thus creating a multistep link from the kinase domain of titin to gene transcription in the nucleus [95]. Prevention of contraction in primary cultures of neonatal rat cardiomyocytes leads to shuttling of SRF out of the nucleus, suggesting that transcription of muscle specific genes by SRF and contractile activity of a muscle cell are tightly linked, which was also observed for the myomasp – SRF signalling axis [58]. Since autophagy adaptors such as p62 and nbr1 as well as the E3 ubiquitin ligase MURF2 are also involved in this signalling cascade, it may affect not only transcription but also protein turnover [95]. There is additional data suggesting that the ubiquitin/proteasome system is built into the M-band. In *C. elegans*, the M-band proteins UNC-96 and UNC-98 were found to interact with CSN-5 [98]. CSN-5 is a component of the COP9 signalosome complex that is found in multiple organisms and regulates protein stability usually via SCF ubiquitin ligases. Knockdown of csn-5 by RNAi resulted in increased levels of UNC-98 [98]. Furthermore, both UNC-89 and obscurin have been reported to play roles in regulating protein turnover via the ubiquitin/proteasome system. Two portions of UNC-89 have been reported to interact with MEL-26, a substrate recognition protein for cullin 3 [99]. Cullins are platforms for assembly of the ubiquitin protein degradation machinery, including E3 ubiquitin ligases. The data support a model in which the interaction of UNC-89 with MEL-26 inhibits the activity of the cullin complex from promoting the ubiquitin-mediated degradation of MEI-1 (katanin), a microtubule severing enzyme, and this is in some way required for proper thick filament organisation. Similarly, Lange and colleagues reported that in the mouse, degradation of sAnk1.5 is dependent upon obscurin, and is promoted by a cullin 3 substrate recognition protein KCTD6 [91].

While kinase domains in obscurin were cloned since the discovery of this giant protein, their functionality and cellular targets, including their ability to auto-phosphorylate are not very well understood [62,100]. Analysis of obscurin orthologues in Drosophila and *C. elegans* however identified several proteins that bind specifically to the kinase region. Sequence analysis of UNC-89 suggests that at least one of the two kinases is most likely catalytically inactive, only permitting scaffolding functions typically associated with pseudo-kinases [101,102]. Elegant studies in *C. elegans* demonstrated further that the lim-domain protein Lim-9 and the phosphatase SCPL-1 (small CTD phosphatase-like-1) associate with one or both of the kinase domains in UNC-89, respectively [103,104]. Loss or overexpression of SCPL-1 affected muscle function or UNC-89 organisation. The human orthologue for SCPL-1 has not yet been identified, although there is a high degree of sequence homology for the phosphatase domain in human SCP1, SCP2 and SCP3 [104]. In contrast, FHL2, a member of the four-and-a-half lim domain protein family that is among the closest human orthologues for Lim-9 in mammals has been shown to localise at M-bands [57], albeit its interaction with obscurin has not been demonstrated.

The kinase domains of invertebrate obscurin from Drosophila were found to provide binding sites for a range of proteins, including bällchen (ball, an active kinase) and MASK (an ankyrin-repeat protein). Depletion of ball or MASK in flight muscles via siRNA resulted in sarcomeric abnormalities, including missing M-bands and either fragmented Z-discs or Z-disc streaming [105]. Although the Drosophila obscurin kinases were not tested for catalytic activity, repeated attempts to test for phosphorylation of identified interaction partners in *C. elegans* were negative [103].

Apart from kinase domains in titin and obscurin, as well as the metabolic enzymes creatine kinase, adenylate kinase and phosphofructokinase, another kinase that was demonstrated to be involved in M-band regulation is cAMP-dependent protein kinase A (PKA). PKA phosphorylation was shown to prevent the binding of myomesin to titin in vitro [106] and phosphorylation of M-protein by PKA abolishes its binding to myosin in vitro [29]. However, PKA has no effect on myomesin binding to obscurin [55]. The cardiomyopathy associated gene CMYA5 (myospryn) that binds to the C-terminus of titin was also shown to interact with PKA [107], however, currently it is not well understood, which role PKA exactly plays in the response to mechanical and humoral stress in the M-band.

As far as the involvement of phosphatases in M-band assembly and turnover is concerned, the strongest candidate is the phosphatase PP2A. PP2A is targeted to this part of the sarcomere together with ankyrin B by obscurin [93]. In *C. elegans* PP2A is important for myofibrillogenesis [108]. Moreover, the B′ regulatory subunit PPTR-2 localises to the M-band by interacting directly with the kinase region of UNC-89 [108]. Also in the mammalian heart there is evidence for a role of PP2A in heart physiology. PP2A is regulated by beta-adrenergic signalling [109], which is well known to be key in heart failure (reviewed in [110]). Transgenic mice that express a dominant negative version of PP2A (A delta 5) succumb to dilated cardiomyopathy [111] and increased activity of PP2A in mice that are deficient for its regulatory subunit B56alpha lead to a decrease in heart rate and conduction defects as well as increased sensitivity to isoproterenol, a beta-adrenergic receptor agonist [112]. While obviously PP2A has a myriad of other targets outside of the M-band such as four key proteins involved in excitation contraction coupling (L-type calcium channel, ryanodine receptor 2, the Na^+^/Ca^++^ exchanger and Na^+^/K^+^ ATPase; reviewed in [113]), it is becoming more and more likely that a delicate balance of phosphorylation and dephosphorylation may also be involved in the regulation of M-band structure and protein-protein interactions.

Besides kinases and phosphatases, the M-band has also been implicated to be an important hub for Rho-associated and Ca^2+^/calmodulin-dependent signalling. Obscurin harbours an IQ-motif in its C-terminus, which was demonstrated to bind to calmodulin, regardless of presence or absence of calcium [61]. While IQ-domains are present in a variety of signalling proteins and may contribute to their activation and functionality, nothing is known about the functions for this domain in obscurin. However, its close proximity to the SH3-DH-PH domain triplet at the obscurin C-terminus may indicate a modulatory function for its Rho-associated signalling. Indeed, detailed molecular analysis of this signalling node in obscurin revealed their direct interaction with RhoA, localising this small GTPase to the sarcomeric M-band [65]. Intriguingly, this interaction is conserved in invertebrates, as the DH-PH region of UNC-89 binds to Rho-1, the *C. elegans* RhoA orthologue [114]. Analysis of obscurin knockout muscle revealed that M-band association of RhoA is completely lost, with RhoA exhibiting a diffuse localisation pattern [91]. In contrast, overexpression of the SH3-DH-PH domains resulted in increased RhoA activity and expression levels, as well as increased downstream signalling through rho kinase (ROCK1) and citron rho-interacting serine/threonine kinase (Stk21) [65].

## 7 Structure determines function of the M-band

**Fig. 5:**
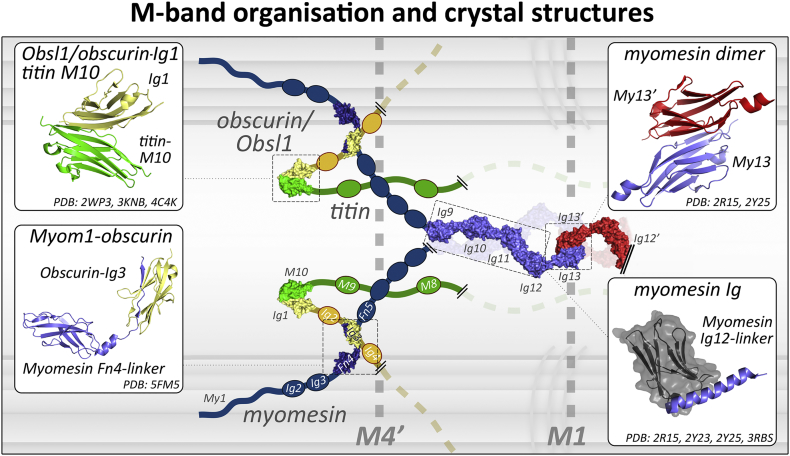
M-band organisation and crystal structures. Correlation of solved crystal structures with a schematic model of the M-band. PDB codes are reported at the bottom of the insets.

The crucial role in the M-band architecture and consequently mechanical stability is played by the three members of the myomesin family, supported by titin, obscurin and Obsl1. At the molecular level the myomesin proteins comprise 13 domains with a common domain layout, i.e. a unique N-terminal domain followed by two Ig, five Fn and another five Ig domains at the C-terminus [8]. However, despite the shared domain layout and the high sequence similarity, they are located in different subregions of the M-band. Similarly, titin and obscurin family members comprise an array of Ig domains that are crossing through the M-band. For titin, its C-terminal 10 Ig domains are localised within the M-band [25]. However, unlike the arrays of titin Ig domains in other parts of the sarcomere, here they are separated by large possibly unstructured sequences with some of them reported to interact through adaptor proteins such as FHL2/DRAL with metabolic enzymes [57]. Less is known about the Ig domains of the obscurin proteins, where at least the first 3 N-terminal of them are assumed to localise within the M-band [55]. During the past decade significant amounts of structural information and biophysical analysis of the interactions between myomesin, obscurin and titin provided a better picture of the M-band architecture and its mechanical properties (Fig. 5).

Crystal structures of the myomesin C-terminus confirmed the antiparallel dimerisation of the domain 13 reported before [27,115] (Fig. 5 top right), further supporting the interpretation that myomesin dimers cross through the M1 line to link the symmetrical parts of the sarcomere [18]. However, more striking was the structure of the C-terminal Ig domains 9–13, revealing an unprecedented Ig-helix pattern, referred to as a hybrid IgH fold [116] (Fig. 5, bottom right). These domains in the myomesin antiparallel dimer are about 36 nm in length, covering most of the total 44 nm distance between the M4′-M4 lines. Atomic force microscopy revealed that this kind or arrangement can be reversibly unfolded on the α-helices that connect the Ig domains to a length of about 2.5 times its original size thus leaving the Ig domains as well as the dimerisation of the protein intact [116,117].

Additional structural evidence on M-band components was recently reported focusing on the M4-M4′ lines where the three major filaments, myomesin, obscurin and titin interconnect. Specifically, the C-terminal Ig domain of titin interacts with the N-terminal Ig domain of obscurin or obscurin-like 1 in an antiparallel conformation, which is conserved in several different examples of Ig domain interactions of the I-set (Fig. 5 top left [118]). On the other side the interaction of obscurin with myomesin-1 is slightly different. Here the third Ig domain of obscurin interacts with a long linker connecting the FnIII domains 4 and 5 of myomesin-1 [[119], [120], [121]] (Fig. 5 bottom left). These interactions support previous evidence that the titin filaments are crossing through the M1 line towards the symmetrical M4 line of the other half of the sarcomere [25]. Similarly, since the N-termini of obscurin filaments are located at the M4 line, they also should be directing towards the M1 line (Fig. 5).

Interestingly the mechanical stability of the obscurin/titin interaction is within a similar range as forces to unfold helices between the C-terminal domains of myomesin (30 pN vs 25 pN), while the disruption of the obscurin/myomesin interaction requires an initial force of about 129 pN, which is similar to the unfolding of Ig domains in the region [116]. The C-terminal domain of myomesin is a very robust domain, but is also protected from dissociation at physiological forces by the alpha-helical linkers leading up to it [117]. Molecular dynamics simulations have suggested that the antiparallel C-terminal dimer outperforms all other myomesin Ig domains mechanically [122]. These results support a basic model where the C-terminal domains of myomesin are the major absorber of mechanical stress that can prevent conformational changes on other interacting sites of the M-band.

Having established a structural/mechanical role for the central part of the sarcomere, further questions arise regarding the role of the additional myomesin isoforms for M-band integrity and how these are related to the specific function of each muscle. Sequence comparison of myomesin with M-protein and myomesin-3 indicates that is quite plausible for all three proteins to hold the same IgH pattern at the C-terminus. It is also striking that even though myomesin and M-protein have a very similar linker between the domains 4 and 5 (identical length and highly conserved amino-acids) there is clear evidence that this linker on M-protein is not interacting with obscurin [121]. Furthermore only myomesin-3 and not M-protein has a confirmed dimerisation ability of the C-terminal domain 13 [8]. Previous work using specific antibodies mapped M-protein to the M1 line [25], but its exact arrangement is unknown at the moment. Given that myomesin-3 is mostly localised at the peripheral M6-M6′ lines a scenario that this protein is building the next transversal level of M-band crosslinks appears to be the most plausible [8].

## 8 Is there life without an M-band?

**Fig. 6:**
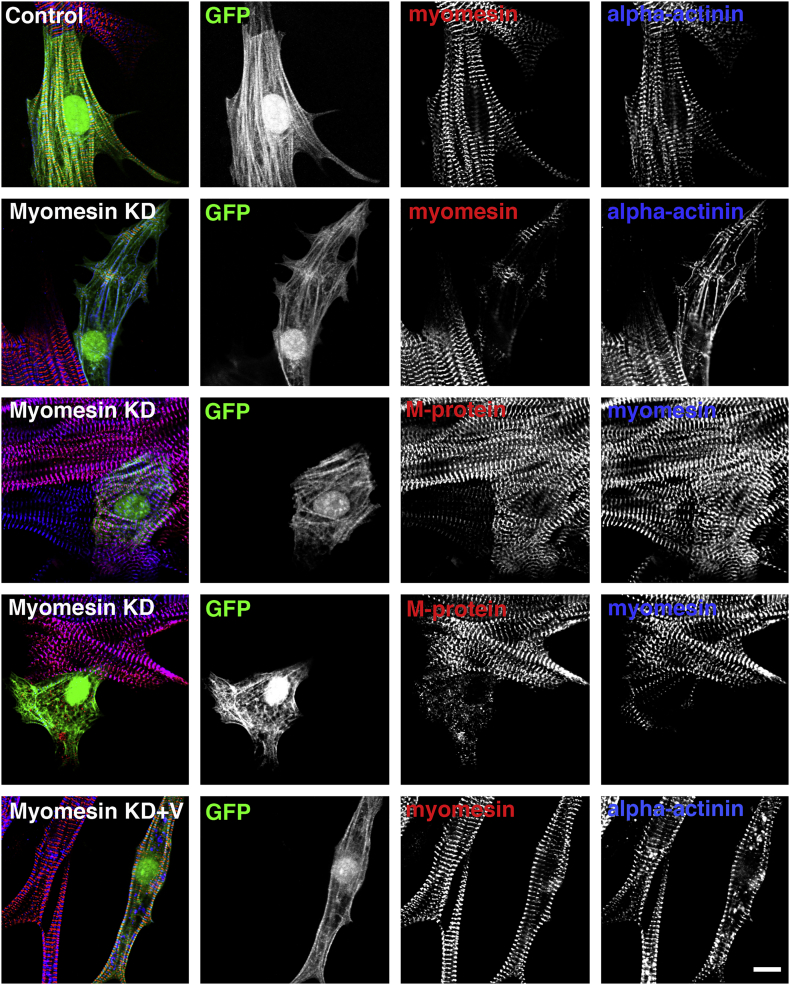
Maintenance of myomesin expression is required for M-band integrity. Confocal micrographs of primary cultures of neonatal rat cardiomyocytes (NRC) either transiently transfected with control plasmid (top row) or myomesin knockdown (KD) plasmid ([55]; four bottom rows) for eight days. The plasmids also encode for GFP to be able to identify the transfected cells (second column). Depletion of myomesin by knockdown in actively contracting NRC leads to disrupted myofibrils (second, fourth row), unless the cardiomyocytes already have started to express M-protein (third row) or contraction is prevented by the addition of verapamil (V) to the culture medium. Antibodies used for immunostaining and their colours in the overlay are indicated in the individual panels. Scale bar = 10 μm.

While the absence of an electron dense M-band in electron micrographs may delude some to the interpretation that this structure is superfluous to sarcomere function [17], all the molecular evidence that is available at present suggests that myomesin-mediated crosslinks between titin and myosin are absolutely crucial. Myomesin accumulations can be detected in the very first sarcomeres that are assembled during myofibrillogenesis [18,[123], [124], [125]]. No animal model(s) that delete one or more members of the myomesin family have been reported. Hence, all loss-of-function studies are based on mutant zebrafish or knockdown experiments of myomesin expression in primary cultures of neonatal rat cardiomyocytes (NRC), which confirm its crucial role in the maintenance of sarcomeres [55]. Short-term (3 days) knockdown of myomesin expression interferes with the integration of obscurin [55], while long-term knockdown (8 days) leads to a disintegration of myofibrils (Fig. 6). Interestingly, the knockdown effect is dependent on the molecular composition of the M-band and also on contractile activity. M-bands in NRC, which already express M-protein in addition to myomesin are protected from disintegration and the phenotype can also not be observed, when contraction is prevented by the Ca^++^ channel blocker verapamil (Fig. 6). More indirect evidence for the relevance of proteins such as myomesin for the maintenance of myofibrils comes from Mef2c knockout mice [126]. Mef2c is a transcription factor that governs among others the expression of myomesin and M-protein and its conditional deletion in skeletal muscle leads to sarcomere fragmentation and perinatal lethality, if a myogenin-Cre strain was used to eliminate the floxed gene [126]. Translation of Mef2 appears to be controlled by muscle activity, via the mTor signalling pathway [127], again linking muscle contraction with downstream transcription of sarcomeric proteins. Titin deletions that eliminate the entire M-band region lead to a failure of myofibrillogenesis [128], while embryos with conditional deletions of two M-band exons of titin (Mex1, Mex2) can initially assemble myofibrils, but display embryonic lethality and sarcomere disassembly, with the severity depending whether an early or later onset strain of Cre was used (alpha-MHC versus M-CK [129]). Interestingly these mice show a broader signal for myomesin compared to their wildtype counterparts, which may indicate that myomesin is targeted via its obscurin/Obsl1 interaction rather than via its conventional binding to titin m4 [130]. Finnish patients that carry a mutation in another exon (Mex6) in the C-terminus of titin, which leads to a loss of C-terminal titin epitopes in histology, show late onset tibial muscular dystrophy [131], potentially due to a loss of binding of titin to obscurin [55]. The muscle phenotype in patients with titin mutations is strongly dependent on the mutational burden, for example homozygous deletions of the C-terminus of titin will lead to early onset myopathy with death due to cardiomyopathy [132,133]. Apart from the evidence from the Mef2c knockout mice discussed above, not much is known about whether M-protein is crucial for muscle function. Patients with an 8p23.2pter deletion, which in addition to several other genes also covers the region that encodes for MYOM2 show developmental delay and a brain phenotype, but their muscles were not investigated [134]. Gene trap experiments that abolished myomesin-3 expression in zebrafish embryos did not result in myofibrillogenesis defects [135], suggesting that there may be a redundancy due to at least five different myomesin genes in zebrafish.

A truncating mutation in MYOM3, which encodes for myomesin-3 in humans, was recently suggested to be a candidate gene for dilated cardiomyopathy [136] and fragments of myomesin-3 in serum were suggested as a biomarker for muscular dystrophy that may even be more reliable than the generally used creatine kinase [137]. Missense mutations in myomesin and obscurin were correlated to a hypertrophic cardiomyopathy phenotype in patients, too [77,138], but clear demonstrations of a functional correlation are still to be carried out. With the advance of next generation sequencing it is very likely that many more missense mutations will be identified in different genes encoding for M-band components, even if the direct correlation between genotype and phenotype will require stringent analysis to differentiate true disease-causing mutations from benign variants [139].

In conclusion, the M-band is central to sarcomere function not only due to its location but also due to its ability to deal with active mechanical stress and to respond in its composition to changes in demand. Its role in (cardio)myopathy is only beginning to be understood, but is expected to expand as more mutations in M-band proteins are detected thanks to next generation sequencing.

## Transparency document

Transparency document. Image 1
